# SG-APSIC1064: The role of infection control activities on healthcare-associated infections during 2017-2021 at intensive care units in Cho Ray Hospital

**DOI:** 10.1017/ash.2023.55

**Published:** 2023-03-16

**Authors:** Dung Tien Phan, Phan Tien Dung, Le Thi Ven, Nguyen Anh Ly, Tran Thi My, Truong Anh Dung, Vu Thi Thuy

**Affiliations:** Cho Ray Hospital, Ho Chi Minh City, Vietnam; Cho Ray Hospital, Ho Chi Minh City, Vietnam; Cho Ray Hospital, Ho Chi Minh City, Vietnam; Cho Ray Hospital, Ho Chi Minh City, Vietnam; Cho Ray Hospital, Ho Chi Minh City, Vietnam; Cho Ray Hospital, Ho Chi Minh City, Vietnam; Cho Ray Hospital, Ho Chi Minh City, Vietnam

## Abstract

**Objectives:** Healthcare-associated infections (HAIs) are one of the greatest challenges and concerns in Vietnam and around the world. Many studies have shown that HAIs may result in an increase in hospital length of stay, antibiotic use, multidrug-resistant organism (MDROs) infections, treatment costs, and mortality. Therefore, in the past 5 years, the Department of Infection Control of Cho Ray Hospital has carried out many infection and prevention control (IPC) activities to reduce the rates of HAI and MDRO infection. We evaluated IPC activities and results achieved in these efforts at Cho Ray Hospital during 2017–2021. **Methods:** We described the implemented IPC activities and retrospectively collected data from HAIs surveillance reports during 2017–2021 for 3 intensive care units (ICUs): ICU-B, ICU-D, and the NICU. **Results:** In the past 5 years, we implemented synchronous IPC activities, including promoting hand hygiene training and surveillance, environmental cleaning surveillance, carrying out improvement projects such as a ventilator-associated pneumonia (VAP) prevention bundle, an MDRO prevention bundle, and an environmental cleaning quality improvement project. Many positive results were achieved, although a slight increase in the HAI incidence occurred in 2021 due to the COVID-19 pandemic. Overall, the hand hygiene compliance rate increased from 49.7% to 83.8%. The rate of HAIs per 1,000 patient days decreased steadily from 5.4 to 2.4. The VAP rate fell from 30.5 to 17.2 per 1,000 patient days, and the central-line–associated bloodstream infection (CLABSI) rate decreased gradually from 5.4 to 2.4 per 1,000 patient days. The catheter-associated urinary tract infection (CAUTI) rate decreased from 2.9 to 0.9 per 1,000 patient days, and the MDRO infection rate decreased significantly from 32.7 to 11.3 per 1,000 patient days. **Conclusions:** The synchronous implementation of HAI prevention bundles promoting hand hygiene and environmental cleaning achieved significant effects in the efforts to decrease HAIs and MDROs in the ICUs of Cho Ray Hospital.

