# SG-APSIC1064: The role of infection control activities on healthcare-associated infections during 2017-2021 at intensive care units in Cho Ray Hospital – CORRIGENDUM

**DOI:** 10.1017/ash.2023.437

**Published:** 2023-09-20

**Authors:** Dung Tien Phan, Phan Tien Dung, Le Thi Ven, Nguyen Anh Ly, Tran Thi My, Truong Anh Dung, Vu Thi Thuy, Phung Manh Thang

In the above published article^1^, the author Phung Manh Thang was accidently omitted from the author list.
